# Optical Coherence Tomography Angiography Imaging to monitor Anti-VEGF treatment of Corneal Vascularization in a Rabbit Model

**DOI:** 10.1038/s41598-019-54171-5

**Published:** 2019-11-26

**Authors:** Kavya Devarajan, Hon Shing Ong, Nyein C. Lwin, Jacqueline Chua, Leopold Schmetterer, Jodhbir S. Mehta, Marcus Ang

**Affiliations:** 1 Singapore Eye Research Institute, Singapore National Eye Center, Singapore, Singapore; 2 Eye-ACP, Duke-NUS Graduate Medical School, Singapore, Singapore; 3 Department of Clinical Pharmacology, Medical University of Vienna, Vienna, Austria; 4 Center for Medical Physics and Biomedical Engineering, Medical University of Vienna, Vienna, Austria; 5 Nanyang Technological University, Singapore, Singapore

**Keywords:** Drug delivery, Diagnostic markers, Experimental models of disease, Preclinical research, Translational research

## Abstract

Optical coherence tomography angiography (OCTA) is a well-established non-invasive retinal vascular imaging technique. It has been recently adapted to image the anterior segment and has shown good potential to image corneal vascularisation. The purpose of the study is to evaluate the usefulness of OCTA to monitor regression of corneal vessels following anti-VEGF (vascular endothelial growth factor) treatment using a previously established corneal vascularisation rabbit model. The regression of vessels following the treatment with aflibercept and ranibizumab anti-VEGFs using both topical instillation and sub-conjunctival injection was quantified using OCTA and compared with ICGA (indocyanine green angiography). Overall vessel density measurements using OCTA showed good correlation (r = 0.988, p < 0.001) with ICGA, with no significant difference between the two treatment groups (p = 0.795). It was also shown that OCTA provided good repeatability outcomes of the quantitative measurements. Using Bland-Altman plots, vessel growth density values between anti-VEGF treatments were compared to control saline group. It was observed that aflibercept provided longer lasting effect than ranibizumab. We also observed that in both drugs, the topical route of administration topical provided longer regression outcomes compared to one-time sub-conjunctival injection. Thereby, with this pilot study, it was demonstrated that OCTA is a reliable imaging technique to follow-up and monitor corneal vascularisation and its treatment quantitatively.

## Introduction

A wide variety of insults to the cornea, ranging from chemical injuries to microbial keratitis can disrupt the corneal vascularity and affect corneal clarity leading to visual impairment^1^. Abnormal corneal angiogenesis, may lead to corneal opacification, which is one of the most common causes of irreversible visual impairment worldwide^2^. Treatment options that have been described include topical corticosteroid^3^, non-steroid anti-inflammatory medications^4^, cyclosporine^5^, photodynamic therapy^6^, laser photocoagulation^7^ and fine needle diathermy^8^. However, none of these options target the molecular mediators of angiogenesis and may provide limited clinical efficiency or undesirable side-effects^9^.

Anti-vascular endothelial growth factor (anti-VEGF) therapies are effective and well-tolerated medications that have revolutionized the treatment of retinal conditions such as neo-vascular age-related macular degeneration and macular oedema in diabetic retinopathy or retinal vein occlusions^10^. The therapy is now considered standard of care in clinical practice for conditions where there is abnormal vasculature in the retina and choroid^11^. Anti-VEGF antibodies are recently being investigated as new promising therapies for corneal vascularization as they suppress angiogenesis by direct VEGF inhibition^12^. The most commonly used drugs in corneal applications have been bevacizumab and ranibizumab. Ranibizumab has been shown to provide better penetration, through the corneal epithelial barrier, than larger biologic agents such as bevacizumab and thus reaching higher therapeutic concentrations in the stroma^13^. From the literature, it is suggested that ranibizumab may be modestly superior to bevacizumab in the treatment of corneal neovascularisation in terms of both on-set of action and degree of efficacy, although direct comparisons have failed to show a clear benefit^14^. Aflibercept, anti-VEGF antibody, has also been recently used for corneal neovascularization, and provides higher binding affinity of VEGF by also interacting with platelet-derived growth factor (PDGF)^13,14^^,^. The tighter binding of the anti-VEGF to the native receptor, contributes it to a longer half-life compared to other anti-VEGFs, that allows for extended dosing intervals^15,16^.

Although there have been a few studies comparing the efficacy of topical and sub-conjunctival anti-VEGF route administration for the treatment of corneal vascularisation, direct comparisons between ranibizumab and aflibercept are lacking^17,18^. Moreover, reliable and objective tools for the imaging of corneal vascularization treatment have not been studied for anti-VEGF therapies. Robust quantitative diagnostic evaluations are of necessity in clinical translational research. Therefore, in order to determine the true superiority of the modes of administration in different drugs, one-to-one comparison studies using quantitative tools need to be evaluated.

We have previously described the use of ASOCTA (anterior segment optical coherence tomography angiography) as a quantitative diagnostic tool for corneal vascularization in a rabbit model, where we compared it to ICGA and slit lamp bio-microscopy, demonstrating good repeatability and better vessel delineation than other conventional techniques^12^. We have also shown that ASOCTA allows quantitative monitoring of vascularized area after antiangiogenic treatment in human subjects^12^. Potential clinical application of the ASOCTA and its advantages in monitoring new vessel development in three dimensions using en-face segmentation has been previously described^19^. However, in order to effectively understand the treatment and its response to corneal vascularization, objective imaging and comparisons of vessel regression or re-growth is required. Thus, this study aims to show the potential of ASOCTA in monitoring vessel regression after treatment of corneal vascularization compared to conventional ICGA.

## Results

**Figure 1:**
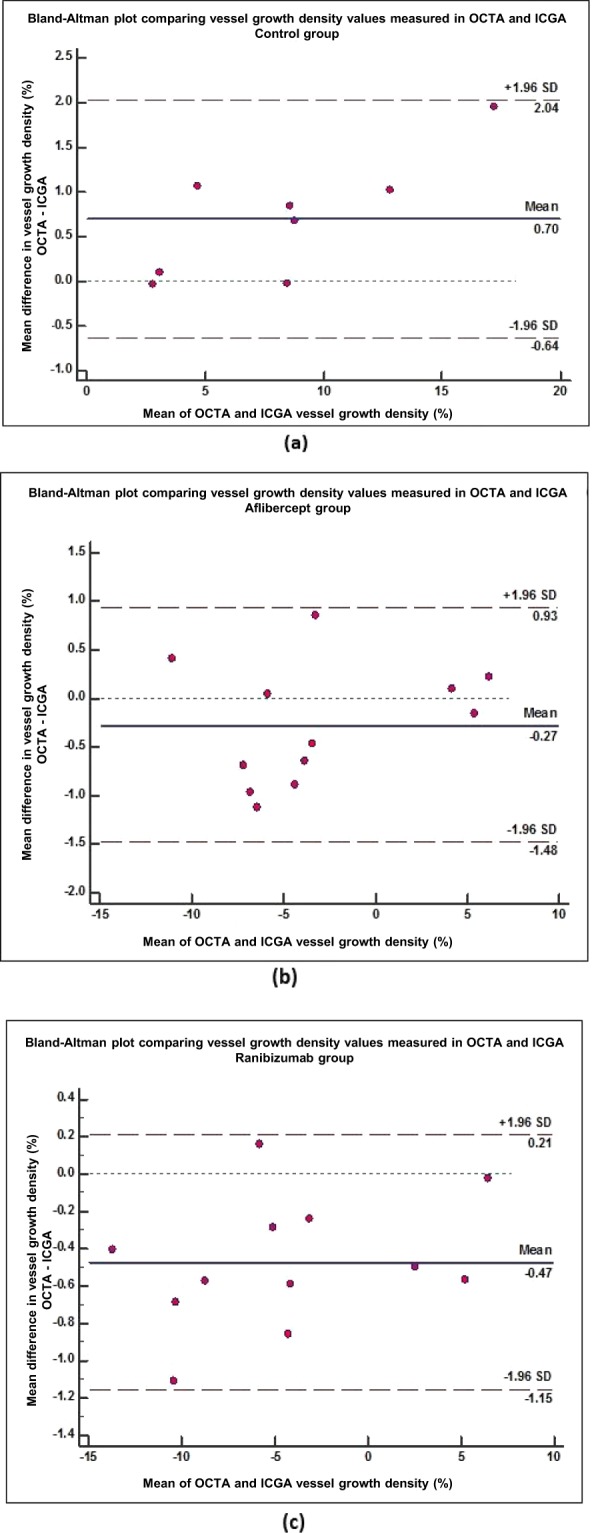
Bland-Altman comparing vessel growth density (%) measurements computed using ICGA and ASOCTA imaging techniques. Mean difference in vessel growth density measurements between the two imaging modalities (y-axis) are plotted against the average measurements taken with the two methods (x-axis). This is compared for each group of drug administration (**a**) Saline control group; Mean difference = 0.70% (95% CI: 0.13 to 1.270) (**b**) Aflibercept treatment group; Mean difference = −0.27% (95% CI: −0.67 to 0.12%) and (**c**) Ranibizumab treatment group; Mean difference = −0.47% (95% CI: −0.69 to −0.25%).

We found good agreement of vessel density measurements between ASOCTA and ICGA, as demonstrated by the Bland-Altman plots of mean vessel growth density (%) values from ASOCTA (1.285 ± 9.248%) and ICGA (1.339 ± 9.248%) within 95% limits of agreement (LOA) – Fig. 1. Furthermore, we found no significant difference in vessel density measurements between both imaging systems (p = 0.795); and a high correlation coefficient (r = 0.988, p < 0.001) was obtained. Good repeatability was also observed between vessel growth density values in OCTA images obtained from the same scan region at one particular time-point (2.35 ± 5.84%), versus between follow-up image scan interest regions (4.54 ± 8.38%), with a high significant difference (p < 0.01).

**Figure 2:**
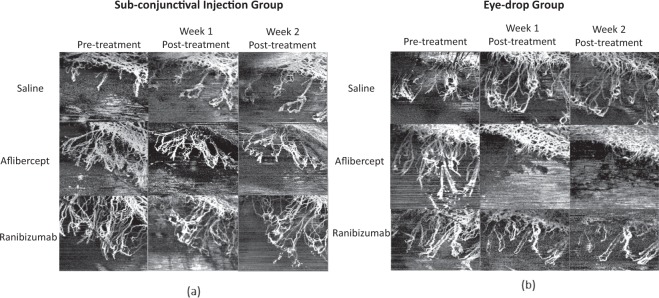
ASOCTA images comparing the different groups of treatment. ASOCTA illustrative images taken at week 1 and week 2 post-treatment are compared to pre-treatment time-point (Week 2 post-suture) in saline, aflibercept and ranibizumab groups.

**Figure 3:**
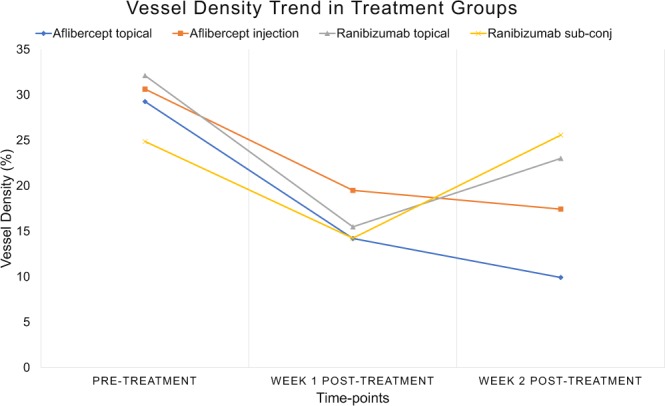
Graph illustrating the trend of vessel density percentage values, from pre-treatment to week 1 and week 2 post-treatment follow-up times. Results showed that significant vessel regression was observed in the week 1 post-treatment time-point with all Anti-VEGF groups as compared to the pre-treatment time-point (p < 0.05) – aflibercept topical instillation (µ_topicalAweek1_ = 14.203 ± 5.7% vs µ_pre-treatment_ = 29.26 ± 2.55%); aflibercept sub-conjunctival injection (µ_injectedAweek1_ = 19.49 ± 2.16%vs µ_pre-treatment_ = 30.63 ± 4.42%); ranibizumab topical instillation (µ_topicalRweek1_ = 15.48 ± 1.86% vs. µ_pre-treatment_ = 32.12 ± 2.98%); ranibizumab sub-conjunctival injection (µ_injectedRweek1_ = 14.23 ± 2.89% vs µ_pre-treatment_ = 24.87 ± 3.89%). At week 2 post-treatment with aflibercept drug administration, the vessel density percentage was reduced and sustained in comparison to pre-treatment imaging in both the sub-conjunctival (µ_injectedAweek2_ = 17.43 ± 4.46% vs µ_pre-treatment_ = 30.63 ± 4.42%; p = 0.099) and topical instillation (µ_topicalAweek2_ = 9.91 ± 1.16% vs µ_pre-treatment_ = 29.26 ± 2.55%; p = 0.0425) routes. With ranibizumab administration, the drug failed to support regression in the Week 2 follow-up imaging time-point and did not provide significant vessel growth density changes in both injection (µ_injectedRweek2_ = 25.57 ± 5.45% vs µ_pre-treatment_ = 24.87 ± 3.89%; p = 0.82) and topical instillation routes (µ_topicalRweek2_ = 23.01 ± 2.27% vs µ_pre-treatment_ = 32.12 ± 2.98%; p = 0.118) with respect to the pre-treatment time-point.

**Figure 4:**
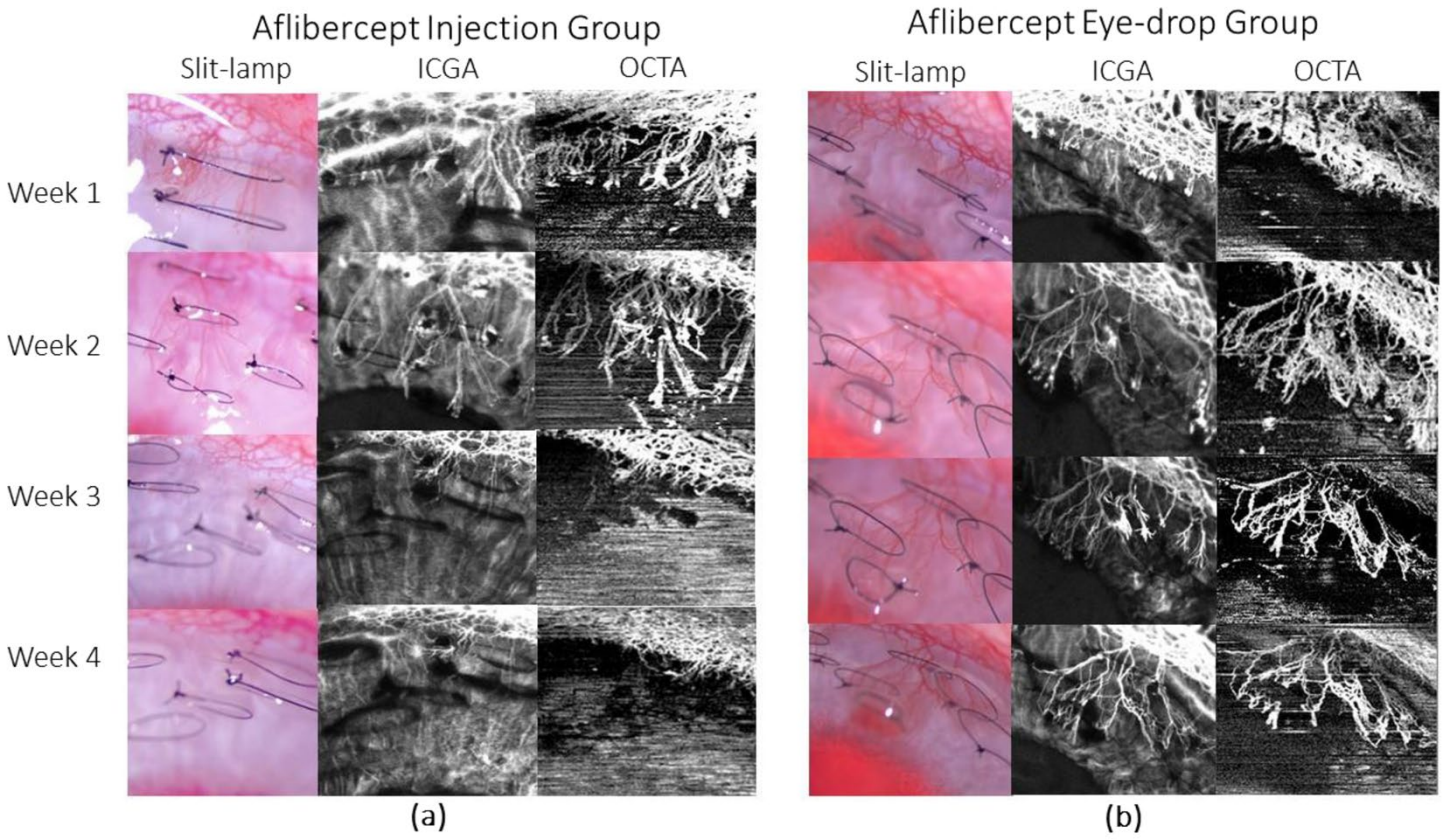
Representative images taken with slit lamp bio-microscopy, ICGA and ASOCTA at the weekly time-points for aflibercept group. At week 3 time-point, the eyes were administered with aflibercept. (**a**) Shows the sequence of images captured for rabbit injected with aflibercept through sub-conjunctival route, whereas (**b**) represents image sequence for rabbit instilled with aflibercept eye-drops.

**Figure 5:**
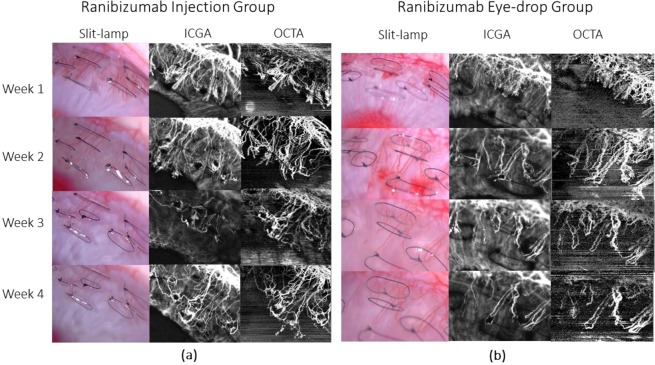
Representative images taken with slit lamp bio-microscopy, ICGA and ASOCTA at the weekly time-points for ranibizumab group. At week 3 time-point, the eyes were administered with ranibizumab. (**a**) Shows the sequence of images captured for rabbit injected with aflibercept through sub-conjunctival route, whereas (**b**) represents image sequence for rabbit instilled with ranibizumab eye-drops.

All vessel growth density values measured at week 1 and week 2 post-treatment using both anti-VEGF drug administrations showed significant reduction as compared to that of the control groups (p < 0.001) – Figs. 2 and 3. The trend of vessel density values measured at the post-treatment time-points as compared to the pre-treatment vascularised state, is graphically illustrated in Fig. 3. At week 1 post sub-conjunctival injection treatment, vessels were seen regressing from the inner fourth row stitches to the second row from the limbus with aflibercept treatment (Fig. 4a), whereas vessels regressed to the third row of stitches in the ranibizumab treatment group (Fig. 5a). Observations at week 2 post-injection, showed corneal vessels regressing further to the second/first row of stitches in the aflibercept group (Fig. 4a). However, with ranibizumab injection, the vessels did not regress and started growing back inwards to the third row of stitches (Fig. 5a). Following eye drop administration for one week, the corneal vessel regression occurred from the fourth row to the second row of sutures in both the aflibercept and ranibizumab experimental groups. After two weeks, both the treatment groups demonstrated further regression, where the aflibercept treatment group relatively showed more regression of vessels (Figs. 4 and 5b).

The routes of drug administration for vessel regression change were compared. Both anti-VEGFs were found to have similar vessel regression differences (p = 0.149) at week 1 (interquartile range; −10.66 to −7.32%) and week 2 post-treatment (interquartile range; −9.87 to −4.48%) with eye drop administration. However, using sub-conjunctival injection there was significantly less vessel density regression at week 2 (interquartile range; −4.84 to −4.47%) as compared to week 1 post-treatment (interquartile range; −10.45 to −7.43%, p = 0.037). Particularly, sub-conjunctival injection with ranibizumab showed significant decline in inhibitory activity in the second week to when angiogenesis returned as compared to the initial treatment week follow-up (inter-quartile range: −13.34% to −9.86% vs. −3.84% to 0.76%, p < 0.01). Aflibercept sub-conjunctival injection at week 2 still provided significant vessel density regression as compared to week 1 (interquartile range; −7.56 to −7.01% vs. −5.84 to −4.96%, p = 0.0495).

## Discussion

Our study, investigating the use of ASOCTA to monitor corneal vascularisation, and its regression after treatment revealed several findings. First, we found that the non-invasive, rapid ASOCTA imaging was able to quantify vessel density change with good repeatability and in good agreement with conventional ICGA (Indo-cyanine Green Angiography). Second, the mean vessel density quantified from ASOCTA was greater than that of the ICGA, (similar to previous studies)^20^, that may be attributed to the greater lateral resolution from ASOCTA compared to ICGA. Thus, we observed that, ASOCTA was useful for serial non-invasive quantification of corneal vascularisation to detect both progression and regression. This enabled us to analyse the vessel growth density values computed from the ASOCTA for comparing different anti-VEGF treatments, using different modes of administration. We also observed that the topical administration of anti-VEGF may have provided a more sustained effect than a single sub-conjunctival injection as vessel regression was similar in both groups in the first week but was sustained in the topical anti-VEGF group due to multiple applications. This showed that with repeated sub-conjunctival injections, an even more sustained effect may be observed. Further, when comparing between the types of anti-VEGFs that were administered, our results suggested that the aflibercept group had a longer period of vessel regression compared to the ranibizumab group in both sub-conjunctival injection and eye drop instillation eyes.

The main aim of this current study was to examine the use of ASOCTA, to monitor development and regression of corneal vascularisation in an animal model. Thus far, ASOCTA studies for corneal vascularisation, have exhibited their diagnostic validity against standard imaging techniques. The ability to segment and visualise three-dimensional OCTA segments in the anterior segment has also been described previously^21^. Furthermore, different algorithm employed by different OCTAs, e.g. SD (Spectral domain) ASOCTA and SSADA (split-spectrum amplitude-decorrelation angiography) ASOCTA, have been compared for their accuracy in quantitative assessments, in corneal vascularisation^12,22^. This study has shown the use of CODAA (Complex OCT signal Difference Analysis Angiography) ASOCTA^23^, for monitoring of the regression of corneal vessels, following treatment. Thereby, in order to assess the reliability, of the values evaluated in this study, repeatability of the vessel density values, of the CODAA ASOCTA, was described. The error in vessel density values captured from the same set of matched images, was found to be significantly lesser than the vessel growth density values taken between weekly follow-up images. In previous studies, repeatability assessments, using SSADA ASOCTA, in rabbit animal models, also suggested the minimal error observed in the repeated scans from the ASOCTA^12^. Our findings also show that CODAA ASOCTA, provides good performance in terms of repeatability and reliability, in both progressive and regressive corneal vasculatures. Further studies are required to compare the vessel regression profiles between different OCTA systems for improving treatment monitoring. Due to the differential wavelength, resolution and speed working ranges, the quantification between different OCTA systems may vary and need to be standardised. This will enable more reliable monitoring of treatment across the different OCTA modalities.

Although administration of anti-VEGF antibodies has been shown to reduce corneal neovascularization, these drugs are yet to be approved for this indication^17,18^. In our study, we observed that current formulations, require repeated treatments to maintain a treatment effect, over time. In this pilot study, we compared the effects of two different drugs, and their modes of administration, to treat corneal vascularisation. Further studies with longer follow-up durations and testing of different drug formulations are required to establish the optimal anti-VEGF treatment regime for corneal vascularisation. Also, the effects of the drug treatment, presented in this study are relevant to newly induced corneal vessels. It is known that anti-VEGF agents are quite effective in occluding actively growing blood vessels, but not in established large vessels^24^. It is also important to consider the advantages and disadvantages between these routes of drug administration when translating to clinical practice. Topically instillation of anti-VEGFs is less invasive but requires frequent dosing. Sub-conjunctival injections though, are more invasive but have effects lasting up to one week with a single dose. In real practice, topical administration of drugs may allow frequent dosing with less side-effects^25^ as compared to repeated weekly injections. However, with the study design, it was estimated that the cost of eye drop instillation costs up to ten times higher than the sub-conjunctival injections for similar effect. Thereby, weekly sub-conjunctival injection may be more cost-effective compared to topical instillation of the drug. In view of the pros and cons from the two routes of anti-VEGF administrations, development of drug delivery devices to administer long-term levels of Anti-VEGFs to corneal tissues need will be considered for the future.

The current study has several limitations. The findings are relevant in the case of optimal operation of the system, where there is control of eye movements and limited motion artefacts present. Repeatability and follow-up analysis of vessel measurements may be less conducive to perform in the case of non-ideal situations, in real-time clinical settings where eye movement artefacts may be prevalent in patients. Hence, future studies, to investigate the clinical usefulness of AS-OCTA, does require further large-scale studies, in patients. Secondly, the algorithm used in the OCTA, was the one commercially available for the posterior segment. With the current segmentation algorithm, the manual correction of the segmentation is still required in minority of the cases. To image the cornea, segmentation and acquisition protocol need to be customised for accuracy. Finally, the effect of signal strength index in quantification using CODAA ASOCTA has not been evaluated. Since poor quality images may have considerable change in the diagnostic value for monitoring treatment, future studies will need to also examine the signal strength indices for reliable measurements.

In summary, we demonstrated for the first time, that ASOCTA is a non-invasive, dye-free and convenient imaging technique to quantitatively monitor corneal neovascularisation treatment. The technology was deemed to be comparable to ICGA in quantifying the vessel growth and vessel regression measurements following anti-VEGF treatments using sub-conjunctival and eye drop instillation of aflibercept and ranibizumab drugs. In both drugs, daily topical instillation of Anti-VEGFs provided a longer lasting effect than one-time sub-conjunctival injection wherein aflibercept provided a longer lasting effect. Anterior-segment ASOCTA will be a useful diagnostic tool for future studies comparing various anti-VEGF drug formulations required to establish the treatment regime for corneal vascularisation.

## Materials and Methods

All animals were treated as per the guidelines of Association for Research in Vision and Ophthalmology’s statement for the Use of Animals in Ophthalmic and Vision Research. Animal work protocols were carried out as approved by the Institutional Animal Care and Use Committee of SingHealth, Singapore. Sixteen healthy adult New Zealand white rabbits of either sex weighing between 2.5–3.5 kg, and of age group 12–15 weeks were housed under standard laboratory conditions at the SingHealth Experimental Medical Centre (Singapore General Hospital, Singapore).

We used a previously established, experimental model of suture-induced corneal vascularisation, where the right eye of each rabbit was subject to corneal suturing^26^. Following the suturing experiment, the eyes were evaluated with slit lamp bio-microscopy (digital slit-lamp camera Righton NS-2D, Tohoku Right Mfg., Miyagi, Japan), ICGA (HRA2, SPECTRALIS® scanning laser angiography, Heidelberg Engineering, Heidelberg, Germany) and CODAA (Complex OCT signal Difference Analysis Angiography) ASOCTA, (Nidek RS-3000) on a weekly basis. Antibiotic eye drops were administered twice a day, during the study period. All surgeries and imaging evaluations were performed under general anaesthesia with xylazine hydrochloride (5 mg/kg intramuscularly; Troy Laboratories, Smithfield, Australia) and ketamine hydrochloride (50 mg/kg intramuscularly; Parnell Laboratories, Alexandria, Australia). At two weeks’ post-surgery, we observed adequate growth of corneal vessels reaching the central cornea in all eyes. In order to not restrict the vessel growth inducing factor from the disease model, the sutures were not removed from the cornea.

At day 15 after corneal neovascularization was induced, treatment was given to the rabbit eyes either through sub-conjunctival administration (n = 8) or topical eye drop instillation (n = 8). Treatment was provided for two weeks. In the first group, the animals were divided into three subgroups based on the specific type of sub-conjunctival injections they were administered with. Each experimental rabbit eye group (n = 3) received either a single dose 0.05 ml sub-conjunctival injection of 0.5 mg ranibizumab (Lucentis; Novartis Pharma AG, Basel, Switzerland) or 0.05 ml sub-conjunctival injection of 2 mg dose aflibercept (Eylea; Bayer Pharma AG; Berlin, Germany). Control eyes (n = 2) received normal saline injections. The second group of rabbit eyes comprised of topical instillation in three subgroups of rabbits depending on the eye-drop that was applied. First subgroup (n = 3) received topical ranibizumab (Lucentis) and the second subgroup (n = 3) received topical aflibercept (Eylea). The last group were controls (n = 2) and received topical normal saline. Each eye received four drops (80 µl = 20 µl*4) of the eyedrop twice daily from day 15 after vascularisation in the cornea was induced, until the last day (day 29) of follow-up.

**Figure 6:**
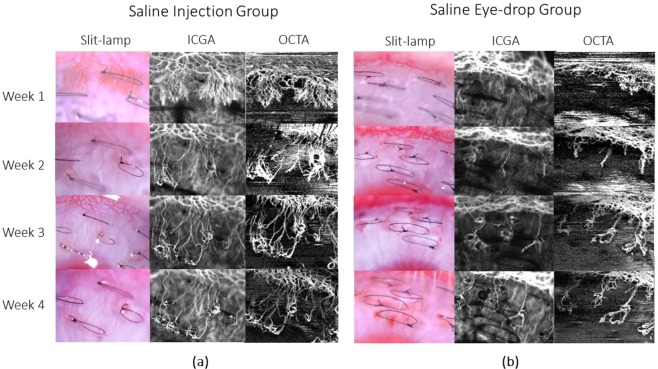
Representative images taken with slit lamp bio-microscopy, ICGA and ASOCTA at the weekly time-points for saline group. At week 3 time-point, the eyes were administered with normal saline. (**a**) Shows the sequence of images captured for rabbit injected with saline through sub-conjunctival route, whereas (**b**) represents image sequence for rabbit instilled with saline eye-drops.

The area of corneal vascularisation was documented using slit lamp bio-microscopy, followed by ASOCTA and then ICGA during each imaging time-point. For ASOCTA acquisition, the anterior segment lens was used with the AngioMacula scan protocol. The eye tracking and autofocus functions were deactivated in the acquisition software, and the lens moved close to the corneal surface before manual adjustments to the Z-motor positioning and focal length were made for precise focus on the B-scan area of interest^12^. Anterior-segment scans centred on the corneal vasculature were obtained at weeks 1 to 4 following the induction of corneal vascularisation. Figures 4–6 show the representative images from the experimental groups taken successively.

**Figure 7:**
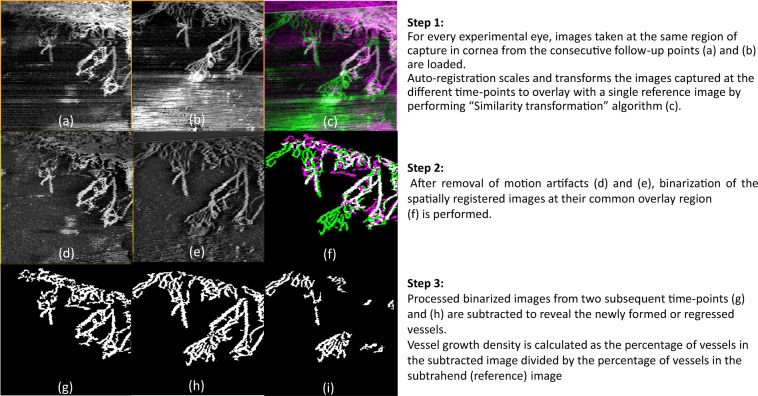
Steps of Image processing to compute vessel growth density percentage in ASOCTA and ICGA images, between consecutive follow-up times. (**a**,**b**) Are examples of ASOCTA images taken at two consecutive follow-up times. (**c**) Shows the overlay of the two raw ASOCTA images after automated registration, where (**b**) is depicted in green and (**a**) is depicted in pink. (**d**,**e**) Are the resulting registered images of (**a**,**b**) after motion artefact removal. Next, binarization of the figures in (**d**,**e**) are performed at their common overlay region and (**f**) represents the binarized overlay. Here the vasculature segmented from (**e**), as processed in (**g**) is color-coded in green, whereas the one from (**d**), processed as (**h**) is color-coded in pink. (**i**) Is the resulting difference in vessels between (**g**,**h**) which represents the vasculature change (growth in this case).

Vessel growth density (%) was computed to determine the growth or regression percentage of vessels imaged by ASOCTA and ICGA at consecutive follow-up scans following similar technique as previously described^20^. The steps of image processing involved are illustrated as Fig. 7. In brief, all the ICGA and ASOCTA images from successive follow-ups for each animal, were overlaid to match areas of same region for image processing. After artefact removal using appropriate filtering techniques, the images were binarized to segment the vessels as white pixels and background as black pixels respectively^20^. Thereafter, image subtraction was performed between successive follow-up scans to determine the vessel growth or vessel regression in each follow up duration. The obtained difference in vessel density percentage between the binary images of consecutive follow-ups was defined as the vessel growth percentage at that time-point. To determine the error in the parameter, repeatability tests were performed in the images taken on the same follow-up on the same animal. Here, the vessel difference value between the matched images were compared with the vessel growth density percentage values between overlay follow-up ASOCTA scans.

Statistical Analysis was performed with MedCalc software (version 18.6) to interpret the vessel growth density (%) values. We used paired two-sample t-test to compare between CODAA ASOCTA and ICGA vessel density measurements and Bland-Altman plots to assess limits of agreement. Paired Mann Whitney tests were used to determine significant differences in the vessel growth density percentage measures between the different treatment groups. In order to evaluate for significant differences between the type of drug and route of drug administration, un-paired t-tests were performed between each experimental group and compared to control groups. P values less than 0.05 were considered significant.

## References

[1] Chang JH, Gabison EE, Kato T, Azar DT (2001). Corneal neovascularization. Curr Opin Ophthalmol.

[2] Whitcher JP, Srinivasan M, Upadhyay MP (2001). Corneal blindness: a global perspective. Bull World Health Organ.

[3] Hos D (2011). Suppression of inflammatory corneal lymphangiogenesis by application of topical corticosteroids. Arch Ophthalmol.

[4] Pakneshan P, Birsner AE, Adini I, Becker CM, D’Amato RJ (2008). Differential suppression of vascular permeability and corneal angiogenesis by nonsteroidal anti-inflammatory drugs. Invest Ophthalmol Vis Sci.

[5] Lipman RM, Epstein RJ, Hendricks RL (1992). Suppression of corneal neovascularization with cyclosporine. Arch Ophthalmol.

[6] Epstein RJ, Hendricks RL, Harris DM (1991). Photodynamic therapy for corneal neovascularization. Cornea.

[7] Park SC, Kim JH (1994). Effects of laser photocoagulation on corneal neovascularization in rabbits. J Refract Corneal Surg.

[8] Faraj LA, Elalfy MS, Said DG, Dua HS (2014). Fine needle diathermy occlusion of corneal vessels. Br J Ophthalmol.

[9] Dastjerdi MH (2009). Topical bevacizumab in the treatment of corneal neovascularization: results of a prospective, open-label, noncomparative study. Arch Ophthalmol.

[10] Korobelnik JF (2014). Intravitreal aflibercept for diabetic macular edema. Ophthalmology.

[11] Brown DM, Regillo CD (2007). Anti-VEGF agents in the treatment of neovascular age-related macular degeneration: applying clinical trial results to the treatment of everyday patients. Am J Ophthalmol.

[12] Ang M (2018). Comparison of anterior segment optical coherence tomography angiography systems for corneal vascularisation. Br J Ophthalmol.

[13] Dastjerdi MH, Sadrai Z, Saban DR, Zhang Q, Dana R (2011). Corneal penetration of topical and subconjunctival bevacizumab. Invest Ophthalmol Vis Sci.

[14] Stevenson W, Cheng SF, Dastjerdi MH, Ferrari G, Dana R (2012). Corneal neovascularization and the utility of topical VEGF inhibition: ranibizumab (Lucentis) vs bevacizumab (Avastin). Ocul Surf.

[15] Papadopoulos N (2012). Binding and neutralization of vascular endothelial growth factor (VEGF) and related ligands by VEGF Trap, ranibizumab and bevacizumab. Angiogenesis.

[16] Thomas M, Mousa SS, Mousa SA (2013). Comparative effectiveness of aflibercept for the treatment of patients with neovascular age-related macular degeneration. Clin Ophthalmol.

[17] Al-Debasi T, Al-Bekairy A, Al-Katheri A, Al Harbi S, Mansour M (2017). Topical versus subconjunctival anti-vascular endothelial growth factor therapy (Bevacizumab, Ranibizumab and Aflibercept) for treatment of corneal neovascularization. Saudi J Ophthalmol.

[18] Chang JH (2012). Corneal neovascularization: an anti-VEGF therapy review. Surv Ophthalmol.

[19] Ang M (2018). Anterior segment optical coherence tomography. Prog Retin Eye Res.

[20] Stanzel TP (2018). Comparison of Optical Coherence Tomography Angiography to Indocyanine Green Angiography and Slit Lamp Photography for Corneal Vascularization in an Animal Model. Sci Rep.

[21] Ang M (2016). En face optical coherence tomography angiography for corneal neovascularisation. Br J Ophthalmol.

[22] Kavya Devarajan, W. D. L. H. S. O., Nyein C.Lwin, Jacqueline Chua, Leopold Schmetterer, Jodhbir S. Mehta and Marcus Ang. Vessel density and En-face segmentation of optical coherence tomography angiography too analyse corneal vascularisation in an animal model. *Eye and Vision* **6** (2019). 10.1186/s40662-018-0128-8 PMC6330743 30656178

[23] Nam AS, Chico-Calero I, Vakoc BJ (2014). Complex differential variance algorithm for optical coherence tomography angiography. Biomed Opt Express.

[24] Feizi S, Azari AA, Safapour S (2017). Therapeutic approaches for corneal neovascularization. Eye Vis (Lond).

[25] de Cogan F (2017). Topical Delivery of Anti-VEGF Drugs to the Ocular Posterior Segment Using Cell-Penetrating Peptides. Invest Ophthalmol Vis Sci.

[26] Perez-Santonja JJ, Campos-Mollo E, Lledo-Riquelme M, Javaloy J, Alio JL (2010). Inhibition of corneal neovascularization by topical bevacizumab (Anti-VEGF) and Sunitinib (Anti-VEGF and Anti-PDGF) in an animal model. Am J Ophthalmol.

